# Neutrophils exhibit distinct phenotypes toward chitosans with different degrees of deacetylation: implications for cartilage repair

**DOI:** 10.1186/ar2703

**Published:** 2009-05-21

**Authors:** Pascale Simard, Hugo Galarneau, Sébastien Marois, Daniel Rusu, Caroline D Hoemann, Patrice E Poubelle, Hani El-Gabalawy, Maria JG Fernandes

**Affiliations:** 1 Centre de Recherche en Rhumatologie et Immunologie, Centre de Recherche du CHUQ-CHUL, boul. Laurier, Québec, G1V 4G2, Canada; 2 Department of Anatomy and Physiology, Université Laval, avenue de la Médecine, Québec, G1V 0A6, Canada; 3 Department of Medicine, Université Laval, avenue de la Médecine, Québec, G1V 0A6, Canada; 4 Department of Chemical Engineering, Institute of Biomedical Engineering, Ecole Polytechnique, boul. Édouard-Montpetit, Montréal, H3C 3A7, Canada; 5 Arthritis Centre, University of Manitoba, Sherbrook Street, Winnipeg, R3A 1M4, Canada

## Abstract

**Introduction:**

Osteoarthritis is characterized by the progressive destruction of cartilage in the articular joints. Novel therapies that promote resurfacing of exposed bone in focal areas are of interest in osteoarthritis because they may delay the progression of this disabling disease in patients who develop focal lesions. Recently, the addition of 80% deacetylated chitosan to cartilage microfractures was shown to promote the regeneration of hyaline cartilage. The molecular mechanisms by which chitosan promotes cartilage regeneration remain unknown. Because neutrophils are transiently recruited to the microfracture site, the effect of 80% deacetylated chitosan on the function of neutrophils was investigated. Most studies on neutrophils use preparations of chitosan with an uncertain degree of deacetylation. For therapeutic purposes, it is of interest to determine whether the degree of deacetylation influences the response of neutrophils to chitosan. The effect of 95% deacetylated chitosan on the function of neutrophils was therefore also investigated and compared with that of 80% deacetylated chitosan.

**Methods:**

Human blood neutrophils from healthy donors were isolated by centrifugation on Ficoll-Paque. Chemotaxis was performed using the chemoTX system. Production of superoxide anions was evaluated using the cytochrome *c *reduction assay. Degranulation was determined by evaluating the release of myeloperoxidase and lactoferrin. The internalization of fluorescently labelled 80% deacetylated chitosan by neutrophils was studied by confocal microscopy.

**Results:**

Neutrophils were dose dependently attracted to 80% deacetylated chitosan. In contrast, 95% deacetylated chitosan was not chemotactic for neutrophils. Moreover, the majority of the chemotactic effect of 80% deacetylated chitosan was mediated by phospholipase-A_2_-derived bioactive lipids. Contrary to the induction of chemotaxis, neither 80% nor 95% deacetylated chitosan activated the release of granule enzymes or the generation of active oxygen species. Despite the distinct response of neutrophils toward 80% and 95% deacetylated chitosan, both chitosans were internalized by neutrophils.

**Conclusions:**

Eighty per cent deacetylated chitosan induces a phenotype in neutrophils that is distinct from the classical phenotype induced by pro-inflammatory agents. Our observations also indicate that the degree of deacetylation is an important factor to consider in the use of chitosan as an accelerator of repair because neutrophils do not respond to 95% deacetylated chitosan.

## Introduction

Osteoarthritis (OA) is characterized by progressive destruction of cartilage in the articular joints [1]. Because it is one of the main causes of disability, this form of arthritis is a burden to both society and the patient. The incidence of OA increases with age. Over 80% of the elderly population exhibits radiographic evidence of OA.

Focal cartilage lesions in humans can be treated by microfracture. This resurfacing procedure, when successful, can re-stabilize the joint and slow the progression of OA. Chitosan was recently shown to promote the regeneration of articular cartilage through the application of an *in situ *solidifying chitosan-glycerol phosphate/blood clot over lesions treated with microfracture [2,3]. Chitosan-glycerol phosphate/blood clots represent a novel articular cartilage repair approach, which has yielded promising results in the clinic [4].

Chitosan is a linear polymer of β (1→4)-linked glucosamine and *N*-acetyl-D-glucosamine residues obtained by the *N*-deacetylation of chitin. Chitosan is biodegradable, non-toxic, and nonimmunogenic [5-7]. The degree of deacetylation (DDA) influences the physical properties of chitosan. As the degree of deacetylation increases, the degree of solubility of chitosan in different solvents decreases and susceptibility to lysosomal biodegradation decreases [8,9]. The chitosan used in the cartilage repair model is of medium viscosity and is 80% deacetylated (80 M). *In vivo*, 50% to 80% deacetylated chitosan is slowly degraded and eventually cleared by enzymatic and cell-based mechanisms [5,10].

The presence of 80 M chitosan over repairing microfracture or microdrill holes is associated with the recruitment of polymorphonuclear neutrophils (PMNs) to the granulation tissue as well as remodeling and revascularization of the damaged trabecular bone, and subsequent formation of more hyaline repair tissue in both rabbit and sheep repair models [2,3,11]. In contrast, few PMNs home to microdrills in the absence of chitosan [11]. Remarkably, PMNs persist in repairing defects for several weeks, in parallel with clearance of the 80 M chitosan particles. Therefore, in contrast to traditional notions that persistence of PMNs in repairing wounds is detrimental to repair, in this cartilage repair model the persistence of PMNs during the first few weeks of repair is related to a more favorable cartilage repair outcome.

To identify the mechanisms through which 80 M chitosan promotes cartilage regeneration in this repair model, the first objective of the present study was to investigate the effect of 80 M chitosan on the function of PMNs. Even though the response of PMNs toward chitosan has been characterized *in vitro *to some extent [12-16], it remains difficult to compare the results between studies and to draw clear conclusions because the chitosan preparations in most studies vary and details on the quality of the chitosan preparations are rarely provided. With regard to the latter, the presence of endotoxins is an important consideration when investigating PMN responses. In the present study, chitosan preparations of medical grade were used. Regarding the former, some studies use chitosan preparations of unspecified DDA whereas other studies use water-soluble chitosan, which does not form a solid implant [16] or semi-crystalline scaffolds [10]. This complicates the interpretation of the results because the degree of DDA is a determining factor for the physical properties of chitosan, and it is not yet established to what extent PMNs respond differently to chitosans of different DDA. Also, it remains to be determined whether the PMN response varies toward chitosan presented as a particulate or a cross-linked scaffold. To optimize the use of chitosan in clinical applications, it is therefore critical to address the effect of DDA on the ability of chitosan to activate PMNs and to compare different preparations of chitosan (for instance, chitosan suspensions versus scaffolds). The second objective of this study was therefore to compare the response of PMNs to two chitosan preparations of a defined DDA, namely 80 M chitosan and 95% deacetylated (95 M) chitosan. The 95 M chitosan was investigated because our preliminary results indicate that PMNs respond differently to chitosan of this DDA *in vivo*.

## Materials and methods

All preparation and incubation procedures were performed under sterile conditions.

### Materials

The two medical grade chitosan preparations (80.6% or 94.6% DDA) used in this study are certified to contain under 0.2% weight/weight protein, <500 EU/g endotoxin, and <10 parts/million heavy metals. To prevent contamination by endotoxin of chitosan solutions, chitosan powder was dissolved in double-distilled water filtered by MilliQ (Millipore, Billerica, MA, USA), at a resistance below 18.2 MΩcm and levels of trace organic compounds below 30 parts/billion with certified 1.0 N HCl, using heat-treated endotoxin-free glassware and stir bars. Chitosan solutions were manipulated under aseptic conditions with laminar flow hoods and dispensed in sterile cryovials with cryo-resistance silicone gaskets for storage at -80°C. The chitosan solutions are of medium viscosity of 1,422 mPa.S for 80% DDA chitosan, termed 80 M (*Mn *= 176 kDa, polydispersity index [PDI] = 1.4), or 2,964 mPa.S for 95% DDA chitosan, termed 95 M (*Mn *= 147 kDa, PDI = 1.6), as previously described by Ma and coworkers [17]. Chitosan solutions were further diluted with sterile double-distilled water to 5 mg/ml or 0.5 mg/ml stock solutions. The DDA of chitosan was provided by the certificates of analysis from the supplier (Bio Syntech Canada, Inc., Laval, QC, Canada).

Rhodamine B isothiocyanate (RITC) was covalently conjugated with each chitosan (RITC-chitosan) to generate either RITC-80 M (80% DDA, number average molecular weight *Mn *= 144 kDa, PDI = 1.3, 0.5% mol/mol RITC/chitosan) or RITC-95 M (95% DDA, *Mn *= 177 kDa, PDI = 1.1, 0.6% mol/mol RITC/chitosan). The DDA of RITC-chitosan derivatives is reported as unchanged, given that the derivatization level was determined as only 0.5% mol RITC/mol chitosan (as reported by Ma and coworkers [17]). Ficoll-Paque and dextran used for the isolation of PMN were obtained from Pharmacia (Kirkland, Québec, Canada) and fetal bovine serum (FBS) as well as RPMI 1640 were purchased from Wisent (St-Bruno, Québec, Canada). Calcein/AM was obtained from Invitrogen (Burlington, Ontario, Canada). Migration was assessed using the ChemoTx system from Neuroprobe (Gaithersburg, MD, USA) and the purified myeloperoxidase (MPO), *O*-dianisidine dihydrochloride, hydrogen peroxide, and cytochalasin B used in the MPO assay were obtained from Sigma-Aldrich (Oakville, Ontario, Canada). Hexadecyltrimethylammonium bromide (HTAB) was purchased from Fluka Chemie GmbH (Buchs, Switzerland). Cytochrome *c *equine heart and pyrrolidine-1 were purchased from Calbiochem (Gibbstown, New Jersey, USA).

### Isolation of polymophonuclear neutrophils

The Institutional Review Board of the Université Laval (Québec, QC, Canada) approved the study, and volunteers signed a consent form. PMNs were isolated as previously described [18]. Briefly, venous blood was obtained from healthy adult volunteers, in accordance with institute-approved protocols, in tubes containing heparin or isocitrate. No difference was observed between the results obtained with these two anticoagulants. After sedimentation of red blood cells in 2% dextran, PMNs were aseptically purified by centrifugation on Ficoll-Paque cushions. Contaminating erythrocytes were removed by hypotonic lysis and PMNs were resuspended in RPMI 1640 supplemented with 0.1% FBS previously decomplemented at 56°C for 30 minutes, except for the chemotaxis experiment (10% decomplemented FBS).

### Chemotaxis

Chemotaxis was measured as described previously [13]. Briefly, PMNs were resuspended in RPMI-1640 and 10% FBS at a concentration of 10^7 ^cells/ml and were pre-incubated with 1 μg/ml calcein/AM at 37°C for 30 minutes in the dark with constant agitation. Cells were washed twice and resuspended in RPMI/10% FBS at 5 × 10^7 ^cells/ml at 37°C. PMN migration was monitored using a 96-well ChemoTx disposable chemotaxis system. The fluorescence of cells in the filters was measured using a microplate fluorescence reader (FL600; Bio-Tek Instruments, Winooski, VT, USA; excitation wavelength 485 nm, emission wavelength 530 nm). Fluorescence was converted to numbers of PMNs based on a standard curve generated by seeding known numbers of PMNs in the bottom of the chamber. The results are expressed as percentage of migrated cells, calculated as the fluorescence of migrated PMNs/fluorescence of 20,000 PMNs/ml × 100, obtained from the standard curve. In some experiments, PMNs were incubated with a final concentration of 0.5 μg/ml pertussis toxin for 90 minutes or with 10^-7 ^mol/l pyrrolidine-1 for 10 minutes at 37°C before the chemotaxis assay.

### Production of superoxide anions by polymorphonuclear neutrophils

Superoxide anion production in response to 80 M and 95 M chitosan was determined using the cytochrome *c *reduction assay, as previously described [19]. Briefly, freshly isolated PMNs were resuspended at a concentration of 1 × 10^6 ^cells/ml in RPMI supplemented with 0.1% decomplemented FBS and cytochrome *c *at a final concentration of 125 μg/ml. The cells were incubated at 37°C for 5 minutes before the addition of the indicated concentrations of 80 M or 95 M chitosan. Cells were incubated for an additional 10 minutes at 37°C and the reaction stopped on ice for 10 minutes. The samples were then centrifuged at 12,000 *g *for 2 minutes at 4°C and the optical density of the supernatants was read at 540, 550, and 560 nm in a spectrophotometer (Milton Roy Spectronic 1001 Plus spectrophotometer, Milton Roy, Rochester, NY, USA). The amount of superoxide produced was calculated using the following formula: A_550 _- ([A_540 _+ A_560_]/2). The absorbance was transformed into the amount of superoxide produced (nmol/10^6 ^neutrophils) by using a conversion factor of 47.4, derived from the molar extinction coefficient of cytochrome *c*.

### Release of myeloperoxidase and lactoferrin by polymorphonuclear neutrophils

Degranulation was determined using the MPO and lactoferrin assay, as described by Bradley and coworkers [20] and Mocsai and colleagues [21], respectively.

For the MPO assay, PMNs (10^7 ^cells/ml) were incubated with 10 μmol/l cytochalasin B, an actin depolymerizing agent that is known to amplify granule exocytosis, for 2 minutes at 37°C and then with the indicated concentrations of 80 M or 95 M chitosan for 30 minutes at 37°C. A negative control with cytochalasin B and a positive control with cytochalasin B + *N*-formyl-methionyl-leucyl-phenylalanine (fMLP; 5 minutes incubation) were also performed. PMNs were then centrifuged for 1 minute at 12,000 *g *and lysed in HTAB lysis buffer (0.5% HTAB, 50 mmol/l K_2_HPO_4 _buffer [pH 6.0]). One hundred microliters of the lysate and cell pellet was mixed with 2.4 ml of a K_2_HPO_4 _buffer (50 mmol/l phosphate buffer [pH 6.0], 0.2 mg/ml o-dianisidine dihydrochloride, and 0.003% hydrogen peroxide) before reading the optical density at 460 nm in a spectrophotometer (Milton Roy Spectronic 1001 Plus spectrophotometer). Purified human MPO (from 0.0625 to 1 U) was used to generate a standard curve. The value '% release of MPO' corresponds to the ratio of the amount of MPO released/the total amount of released and cellular MPO.

For the lactoferrin assay, PMNs (5 × 10^5 ^cells/ml) were incubated as described above for the MPO experiments. The release of lactoferrin was determined by enzyme-linked immunosorbent assay [21]. Supernatants were diluted 10-fold and 100-fold in 50 mmol/l CO_2_/HCO_3 _buffer (pH 9.6) and 100 μl of the diluted supernatants or of known concentrations of human lactoferrin were added to 96-microwell plates (Nalge Nunc International, Rochester, NY, USA) and incubated overnight at 4°C. Nonspecific binding sites were blocked with phosphate-buffered saline supplemented with 0.5% bovine serum albumin and 0.5% Tween-20 overnight at room temperature. One hundred microliters of rabbit anti-human lactoferrin antibody (1:500 dilution of 17.05 mg/ml stock) was then added to each well and incubated for 2 hours. One hundred microliters of the secondary antibody, peroxidase-conjugated anti-rabbit antibody (1:40,000 dilution), was then added and incubated for 30 minutes. Each of the above steps was performed at room temperature and between each step the plates were repeatedly washed with 1× Tris-buffered saline/0.1% Tween-20. Tetra-methyl-benzidene was added before stopping the reaction with 50 μl of 1 mol/l sulfuric acid. Absorbance was read at 450 nm with a microplate reader, and the lactoferrin concentration was calculated using the standard curve.

### Internalization of 80 M and 95 M chitosan by polymorphonuclear neutrophils

Freshly isolated PMNs were resuspended in RPMI supplemented with 0.1% decomplemented autologous serum, pre-stained with 1 μg/ml calcein/AM for 30 minutes at 37°C, and then incubated with 30 μg/ml of RITC-80 M or RITC-95 M for 3 hours at 37°C. PMNs were then centrifuged for 2 minutes at 1,500 *g *at room temperature and plated on a glass slide coated with 100% decomplemented autologous serum. Slides were coated with autologous serum that was prepared by centrifuging clotted blood for 15 minutes at 700 *g *at room temperature and decomplemented for 30 minutes at 56°C to avoid activation of PMNs by the glass surface of the slide. PMNs were visualized live at 37°C in an environment chamber with 5% CO_2 _through a spinning disc confocal microscope equipped with a 63× objective (Quorum Spinning Disc Wave FX, ON, Canada). The index of internalization of chitosan was calculated as the percentage age of PMNs that internalized any visually detectable quantity of RITC-chitosan. One hundred cells were observed for each experimental condition.

### Interaction of 80 M and 95 M chitosan with monocytes, granulocytes, and lymphocytes in whole blood

The interaction of RITC-80 M and RITC-95 M chitosan with blood cells was determined by flow cytometry. Blood samples were first treated to eliminate erythrocytes by lysis, as described by Desmeules and coworkers [22], and were then stimulated for 30 minutes at 37°C with 5 μg/ml RITC-80 M. Chitosan binding to cells was visualized using FACScan flow cytometry. The binding index was calculated as fluorescence units of a cellular population incubated with RITC-80 M chitosan/fluorescence units of a cellular population incubated in the same volume of the diluent (double-distilled water).

### Statistical analysis

Results are expressed as means ± standard error. Statistical analyses were performed using GraphPad Instat 3.0 (GraphPad Software, Inc., San Diego, CA, USA). Comparisons made between two groups were analyzed with the unpaired Student's *t*-test. Comparisons made between two or more groups were analyzed by one-way analysis of variance and the Tukey-Kramer *post hoc *test. *P *< 0.05 was regarded to indicate statistical significance.

## Results

### The chemotactic effect of 80 M and 95 M chitosan on polymorphonuclear neutrophils

**Figure 1:**
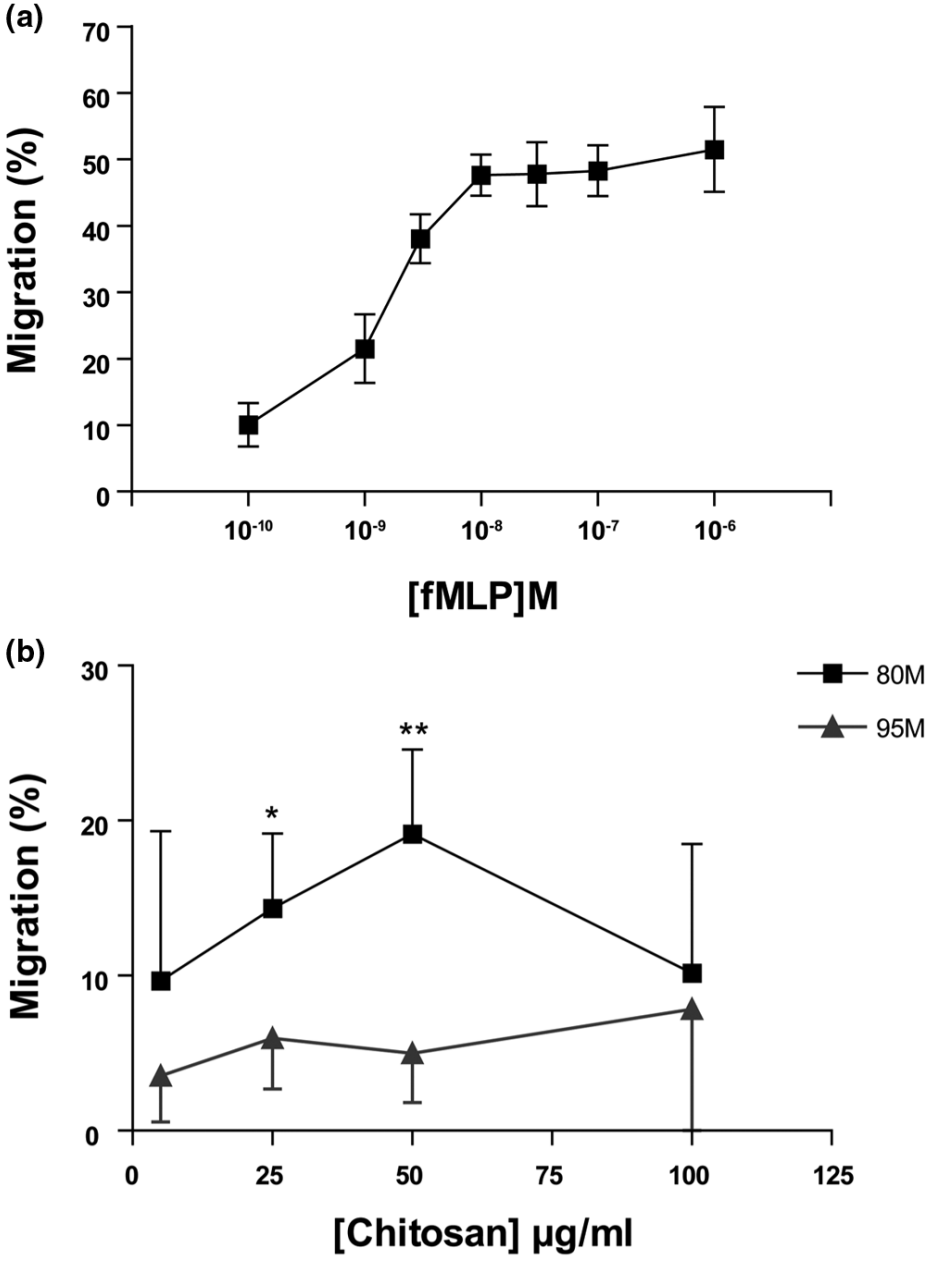
The chemotactic effect of chitosan on PMNs. Freshly isolated polymorphonuclear neutrophils (PMNs) were pre-stained with 1 μg/ml calcein-AM and seeded on a polycarbonate filter placed above a well containing **(a) ***N*-formyl-methionyl-leucyl-phenylalanine (fMLP; positive control) or **(b) **80% deacetylated (80 M; black line) or 95% deacetylated (95 M; gray line) chitosan. PMNs were allowed to migrate for 1 hour before assessing migration, as described in 'Materials and Methods'. Percentage migration of PMNs = the fluorescence of migrated PMNs/fluorescence of 20,000 PMNs/ml × 100, obtained from the standard curve. Results are presented as mean ± standard error. *P *values from Student's two-tailed unpaired *t*-test: *P = 0.01, fluorescence of PMNs migrated toward 25 μg/ml 80 M chitosan versus fluorescence of PMNs migrated toward RPMI (Ctrl-); **P = 0.004, fluorescence of PMNs migrated toward 50 μg/ml 80 M chitosan versus fluorescence of PMNs migrated toward RPMI. The positive control is the fMLP curve. This figure represents the results of three independent experiments.

Before we conducted the study, we verified that we could reproduce the chemotactic activity of 80 M chitosan toward PMNs observed *in vivo*, in the cartilage repair model, with an *in vitro *chemotaxis assay. Briefly, isolated PMNs were labeled with calcein/AM and the chemotactic activity of 80 M chitosan was determined using the ChemoTx chemotaxis system – a transwell migration assay. We provide direct evidence that under our experimental conditions the 80 M chitosan preparation was chemotactic for PMNs (Figure 1).

Chitosan preparations composed of chitosan of varying degrees of DDA, greater than 80%, have been reported to be chemotactic for PMNs *in vitro *and *in vivo *[12]. It is not clear from these studies, however, whether the chemotactic activity of chitosan is dependent on the degree of DDA. To determine whether the chemotaxis of PMNs toward chitosan is dependent on the degree of DDA, a similar chemotaxis experiment was performed with 95 M chitosan. In contrast to 80 M chitosan, 95 M chitosan was not chemotactic for PMNs under the same experimental conditions (Figure 1). These results not only confirmed the potential of 80 M chitosan to attract PMNs but also indicated that the degree of acetylation of chitosan affected its chemotactic activity toward PMNs.

### Mediator(s) of the chemotactic effect of 80 M and 95 M chitosan on polymorphonuclear neutrophils

**Figure 2:**
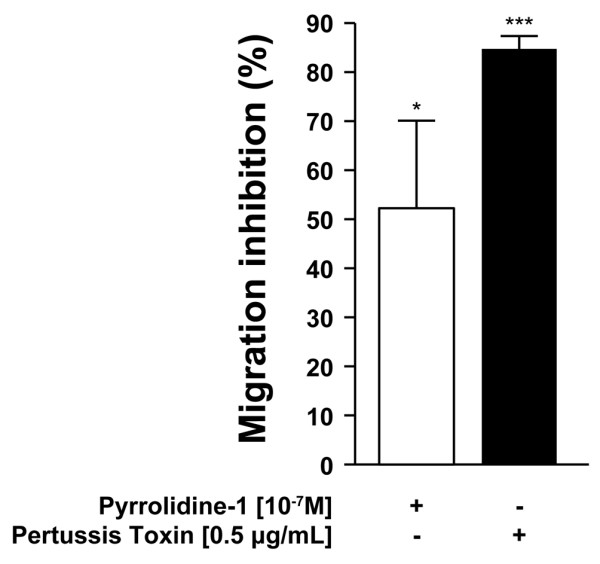
The effect of inhibitors on PMN chemotaxis towards chitosan. Freshly isolated polymorphonuclear neutrophils (PMNs) were resuspended in RPMI 1640 supplemented with 0.1% decomplemented fetal bovine serum, pre-stained with 1 μg/ml calcein-AM for 30 minutes at 37°C and incubated with 0.5 μg/ml pertussis toxin for 90 minutes and seeded on a polycarbonate filter above a well containing 50 μg/ml 80% deacetylated (80 M) chitosan. Alternatively, PMNs were incubated with 10^-7 ^mol/l pyrrolidine for the last 10 minutes of the incubation with calcein-AM. Chemotaxis was performed as described in Figure 1. The percentage inhibition of migration corresponds to the fluorescence of PMNs incubated with the inhibitors that migrated toward 80 M chitosan versus fluorescence of PMN incubated in media that migrated toward 80 M chitosan. This figure represents the results of at least three independent experiments. *P = 0.02 and ***P = 0.0001.

We then investigated the molecular mechanism through which 80 M chitosan induces chemotaxis of PMNs. Because neutrophils are a major source of the strong chemotactic mediators leukotriene B_4 _(LTB_4_) and platelet-activating factor (PAF), we studied the activation of this metabolic pathway in response to 80 M chitosan. LTB_4 _is generated by the oxygenation of arachidonic acid by a 5-lipoxygenase. Arachidonic acid becomes available to 5-lipoxygenase once it is released from 1-*O*-alkyl-2-acyl-glycerophosphocholine by cytosolic phospholipase A_2_-α (cPLA_2_-α) that also releases lyso-PAF simultaneously [23]. To determine whether these phospholipid-derived metabolites are responsible for the chemotactic activity of 80 M chitosan toward human PMNs, the effect of pyrrolidine-1, an inhibitor of cPLA_2_-α, on the chemotaxis of PMNs induced by 80 M chitosan was determined. Briefly, PMNs were pre-incubated with pyrrolidine-1 and then allowed to migrate toward 80 M chitosan. Pyrrolidine-1 decreased the chemotaxis of PMNs toward 80 M chitosan by 50% (Figure 2). These findings indicate that arachidonic acid metabolites are responsible, at least in part, for the chemotactic activity of 80 M chitosan toward PMNs. As a general rule, PMN chemotactic factors (for example, fMLP, interleukin-8, PAF, and LTB_4_) bind G-protein-coupled receptors. The activation of these G-protein-coupled receptors can be inhibited by pertussis toxin. We provide direct evidence that pertussis toxin significantly inhibited the chemotaxis of PMNs toward 80 M chitosan by 80% (Figure 2).

### Production of superoxide anions and degranulation by polymorphonuclear neutrophils in response to 80 M and 95 M chitosan

**Figure 3:**
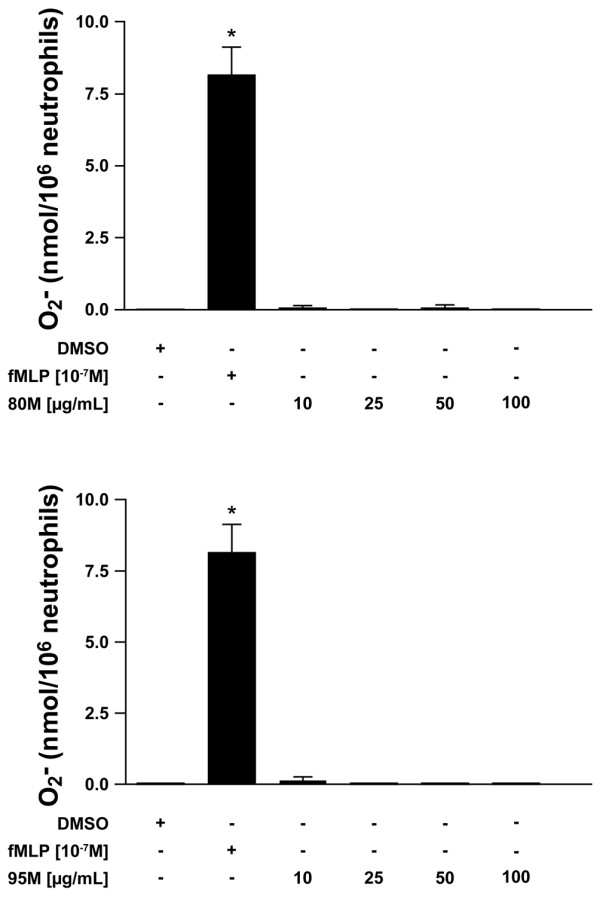
Production of superoxide anions by PMN in response to chitosan. Superoxide anion production was determined using the cytochrome *c *reduction assay. Freshly isolated polymorphonuclear neutrophils (PMNs) resuspended in RPMI 1640 supplemented with 0.1% decomplemented fetal bovine serum were incubated with the indicated concentrations of 80% deacetylated (80 M) or 95% deacetylated (95 M) chitosan for 10 minutes at 37°C. Results are presented as mean ± standard error. The difference from the negative control is statistically significant: *P < 0.001 (Tukey-Kramer test). The negative control = PMNs incubated in Hanks' Balanced Salt Solution supplemented with 0.1% decomplemented fetal bovine serum and incubated with diluent (dimethyl sulfoxide [DMSO]). This figure represents the results of at least three independent experiments. fMLP, *N*-formyl-methionyl-leucyl-phenylalanine.

The mechanisms by which PMNs are thought to impair healing include the production of reactive oxygen species and the release of granule contents. Because PMNs promote wound healing and cartilage regeneration in the presence of 80 M chitosan, it is of interest to determine whether – in the presence of 80 M chitosan – PMNs produce these microbicidal substances. PMNs produce superoxide in response to fMLP, a bacterial-derived antigen (Figure 3). In contrast to the large superoxide burst observed in response to fMLP, neither 80 M (Figure 3a) nor 95 M (Figure 3b) chitosan induced the release of superoxide by PMNs at all of the concentrations of chitosan tested (from 10 to 100 μg/ml). The amounts of superoxide released by PMNs incubated with 80 M or 95 M chitosan were comparable to those of the negative control.

**Figure 4:**
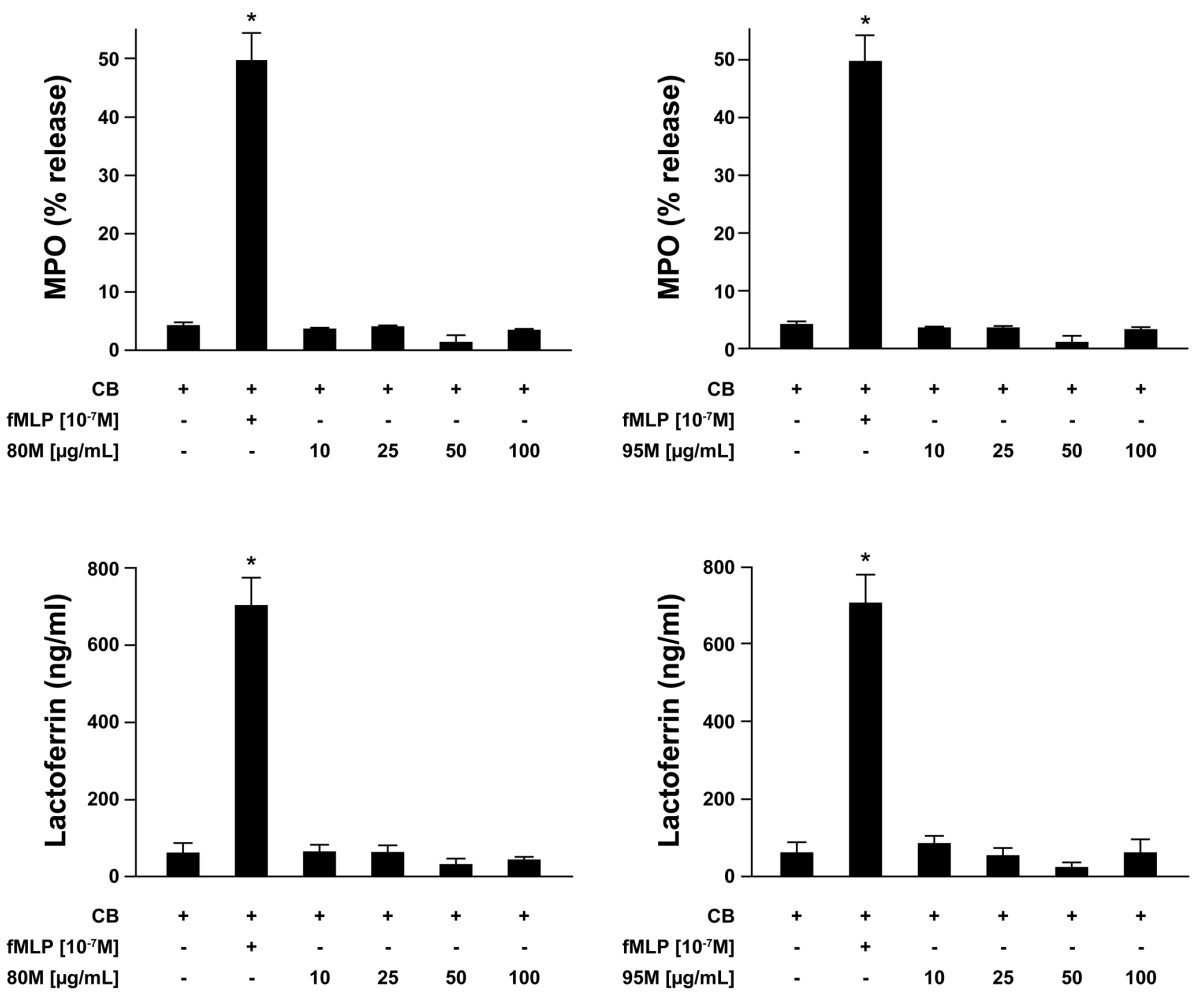
Release of myeloperoxidase and lactoferrin by PMNs in response of chitosan. Degranulation was determined using the **(a, b) **myeloperoxidase (MPO) and **(c, d) **lactoferrin assay, as described in 'Materials and Methods'. Freshly isolated polymorphonuclear neutrophils (PMNs) resuspended in RPMI 1640 supplemented with 0.1% decomplemented fetal bovine serum were treated with cytochalasin B and further incubated with the indicated concentrations of 80% deacetylated (80 M; panels a and c) or 95% deacetylated (95 M; panels b and d) chitosan for 30 minutes at 37°C. The quantity of MPO released is expressed as '% MPO', which corresponds to the ratio of the amount of MPO released/total amount of cellular MPO. The amount of lactoferrin released is expressed in ng/ml. Results are presented as mean ± standard error. The difference from the negative control is statistically significant: *P < 0.001 (Tukey-Kramer test). The negative control = PMNs incubated in Hanks' Balanced Salt Solution supplemented with 0.1% decomplemented fetal bovine serum and incubated with cytochalasin B. This figure represents the results of at least three independent experiments. fMLP, *N*-formyl-methionyl-leucyl-phenylalanine.

To determine whether 80 M and 95 M chitosan induce PMNs to degranulate, the release of the contents of the primary and secondary granules was determined by measuring the amount of MPO and lactoferrin released. When incubated with 10 to 100 μg/ml 80 M or 95 M chitosan, the amounts of MPO and lactoferrin released into the media by PMNs were negligible (Figure 4). Together, the above observations indicate that the effects of 80 M chitosan on PMNs are not associated with the release of granule substances from PMNs.

### The interaction of 80 M and 95 M chitosan with polymorphonuclear neutrophils

**Figure 5:**
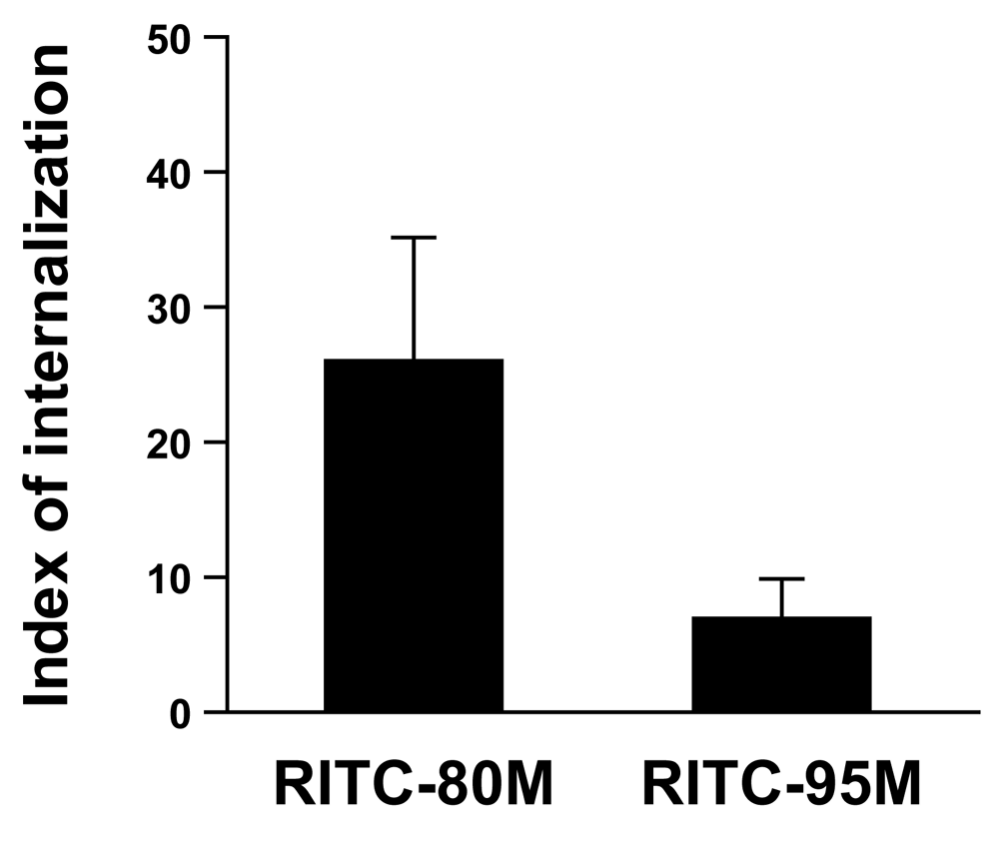
The internalization of chitosan by PMNs. Freshly isolated polymorphonuclear neutrophils (PMNs) were resuspended in RPMI 1640 supplemented with 0.1% decomplemented fetal bovine serum, pre-stained with 1 μg/ml calcein-AM for 30 minutes at 37°C and incubated with 100 μg/1 × 10^6 ^cells rhodamine B isothiocyanate (RITC)-zymosan for 1.5 hours (a positive control), 15 μg/ml RITC-80% deacetylated (80 M) or RITC-95% deacetylated (95 M) chitosan for 3 hours at 37°C. PMNs were then centrifuged and plated on a slide coated with 100% decomplemented autologous serum and visualized by live confocal microscopy. The index of internalization of chitosan by PMNs was calculated as the percentage of cells that internalized RITC-chitosan. Results are presented as mean ± standard error. This figure represents the results of three independent experiments.

The difference in chemotactic activity between 80 M and 95 M chitosan toward PMNs may be due to the inability of PMNs to bind and/or internalize 95 M chitosan. The binding and internalization of RITC-80 M and RITC-95 M chitosan by PMNs was investigated by live cell confocal microscopy. Live cell imaging revealed that PMNs internalized both RITC-80 M and RITC-95 M chitosan in the presence of decomplemented serum, although internalization was much greater for fluorescent zymosan under similar conditions (Figure 5).

**Figure 6:**
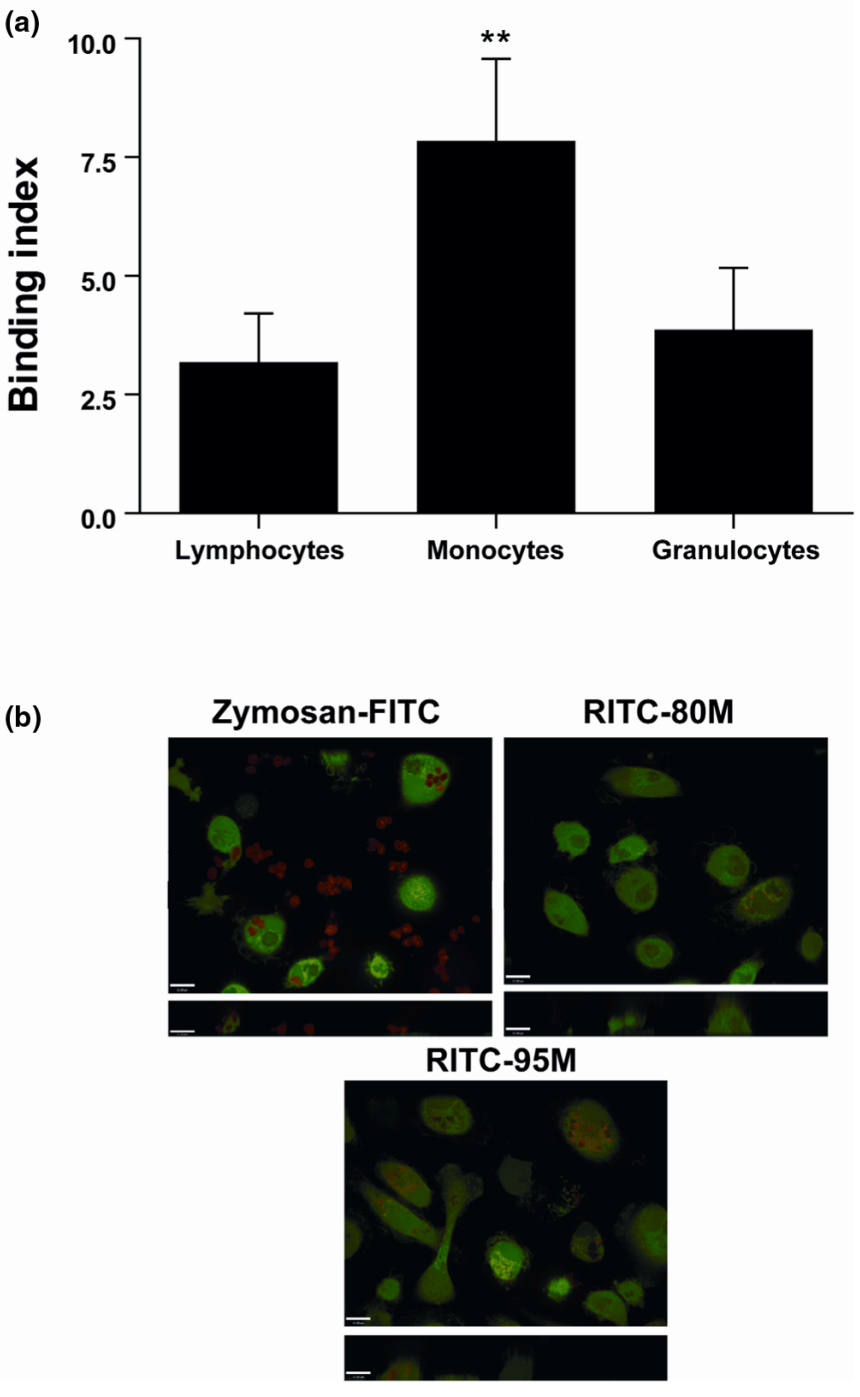
The interaction of chitosan with monocytes, granulocytes, and lymphocytes in whole blood. **(a) **Whole blood was incubated for 30 minutes with 5 μg/ml rhodamine B isothiocyanate (RITC)-80% deacetylated (80 M) chitosan at 37°C for 30 minutes before analysis by flow cytometry. The binding index was calculated as fluorescence units of each leukocyte population incubated with RITC-80 M chitosan/fluorescence units of leukocyte population in the absence of RITC-chitosan. Results are presented as mean ± standard error. *P *values from Student's two-tailed unpaired *t*-test: **P < 0.005 versus autofluorescence for each leukocyte population. **(b) **Macrophages were seeded on glass slides (2 × 10^6 ^cells/ml) in RPMI 1640 supplemented with 0.1% decomplemented fetal bovine serum, pre-stained with 1 μg/ml calcein AM for 30 minutes at 37°C, and incubated with 100 μg/1 × 10^6 ^cells RITC-zymosan for 1.5 hours (a positive control), 15 μg/ml RITC-80 M, or RITC-95% deacetylated (95 M) chitosan for 3 hours at 37°C. Macrophages were then visualized live through a spinning disc confocal microscope with a 63× objective. The top panels are images taken in the X-Y plane and the lower panels are images taken in the X-Z plane. This figure represents the results of three independent experiments.

Because all of the white blood cells are present at the microfracture sites and could be involved in the effects of chitosan on cartilage repair and wound healing, we also investigated the ability of 80 M chitosan to interact with other leukocytes. To observe the interaction of 80 M chitosan with leukocytes with the same differential ratio in which these cells normally co-exist, this analysis was performed in whole blood devoid of erythrocytes. Flow cytometry analysis of leukocytes in whole blood revealed that a greater amount of RITC-80 M chitosan associates with monocytes than granulocytes and lymphocytes (Figure 6a). Confocal microscopy revealed that monocytes readily internalize large amounts of RITC-80 M and RITC-95 M chitosan (Figure 6b).

## Discussion

Novel therapeutic modalities that promote cartilage regeneration have the potential to delay significantly the progression of OA in patients who develop focal lesions. We therefore investigated some of the molecular mechanisms involved in the clinically beneficial effects of 80 M chitosan, which was recently shown to promote cartilage regeneration in both large and small animal cartilage repair models [2,3]. In recent years evidence has accumulated that the PMN is more than just a leukocyte that phagocytoses foreign antigens. PMNs differentiate into dendritic cells [24] and have the capacity to modulate the adaptive immune response. The PMN therefore adopts different phenotypes that are determined by its environment. In this light, the response of PMNs to 80 M chitosan was investigated to identify the characteristics of the phenotype of PMNs that promotes cartilage regeneration. We report that 80 M chitosan selectively activates a subset of PMN functional responses.

The distinct phenotype of PMNs in response to 80 M chitosan is characterized by PMN chemotaxis and the absence of the production of superoxide and degranulation. In comparison, fMLP, a bacterial-derived peptide, is not only chemotactic for PMNs but also stimulates them to produce superoxide and degranulate. These observations strongly suggest that the PMN phenotype in the presence of 80 M chitosan promotes repair due, at least in part, to the lack of superoxide production and PMN degranulation. Our data agree with a recent report indicating that water-soluble chitosan oligomers suppress the capacity for PMNs to respond to phorbol myristate acetate [16].

Because 80 M chitosan is chemotactic for PMNs, it must interact at the surface of PMNs to elicit a chemotactic response. The majority of chemotactic factors mediate their effect through G-protein-coupled receptors. To determine whether this applies to 80 M chitosan, we assessed the effect of pertussis toxin on 80 M chitosan-induced chemotaxis of PMN. Pertussis-toxin inhibited PMN chemotaxis by 80%, implicating a G-protein-coupled receptor. The mechanism through which 80 M chitosan activates a G-protein-coupled receptor remains to be determined. It was previously found that conditioned media from canine PMNs stimulated with >80% DDA chitosan particles promoted chemotaxis of neutrophils [14]. We provide evidence, using a specific cPLA_2_-α inhibitor, that phospholipid-derived mediators, possibly the chemotactic factors LTB_4 _and PAF, are involved in the direct chemotactic activity of human PMNs toward a pure and sterile 80 M chitosan preparation. This is the first study to demonstrate that such lipid mediators contribute to half of the chemotactic activity of human PMNs toward chitosan. The cPLA_2_-α inhibitor pyrrolidine-1 inhibited the chemotaxis of PMNs by 50%. Moreover, the inhibition of chemotaxis by 80% in the presence of pertussis toxin suggests that additional chemotactic agents acting through G-protein-coupled receptors participate in the chemotaxis of PMNs toward chitosan. Further investigation is required to characterize fully the molecular mechanisms that are involved in 80 M chitosan-induced chemotaxis of human PMNs.

Having characterized the response of PMNs toward 80 M chitosan, we conducted similar experiments with 95 M chitosan because we had observed a distinct response of PMNs toward 95 M chitosan *in vivo*. This is the first report on the effect of 95 M chitosan on PMN effector functions. Chitosan 95 M (95% glucosamine, 5% *N*-acetyl glucosamine) was unable to induce chemotactic activity, superoxide production, or the release of granule contents by PMNs. The lack of chemotactic activity of 95 M chitosan toward PMNs was not due to an effect on the viability of PMNs (data not shown). The percentage DDA of chitosan is therefore a determining factor for the activation of PMNs by chitosan and potentially for the therapeutic use of chitosan. Our findings indicate that chitosans in the range from 80% to 95% DDA can elicit quite different biologic responses, and highlight the importance of defining the DDA level when conducting biologic assays. Some of the differential responses could be related to the very low solubility of 95% DDA chitosan at neutral pH [8]. In the light of these findings, it is of interest to investigate fully the effect of chitosan with other percentages of DDA on PMNs to determine whether there is a percentage DDA that induces maximal chemotactic activity in PMNs and consequently an optimal therapeutic effect.

Another parameter that may modify the response of PMNs to chitosan is the form in which the chitosan is used. Vandevord and coworkers [6] reported that a chitosan scaffold made with chitosan of 92% DDA is chemotactic for PMN *in vivo*. Because 92% DDA chitosan is structurally more similar to 95 M than to 80 M chitosan, our data indicate that PMN migration to 92% DDA chitosan should be quite modest. The discrepancy between this previous observation and our findings could potentially be explained by the fact that the scaffold used in the *in vivo *study was prepared by coating polytetrafluoroethylene tubes with 92% DDA chitosan, and did not employ pure chitosan. It will be of therapeutic interest to determine how differently PMNs react to chitosans of the same percentage DDA of different structural forms – suspension versus scaffold.

It is generally accepted that PMN phagocytose chitosan, but no microscopy studies have been performed to demonstrate that PMNs indeed internalize chitosan. This is a relevant question because PMNs can respond to foreign material without necessarily internalizing it. We provide direct evidence that PMNs can internalize 80 M chitosan without stimulating degranulation. Around 10% of PMNs internalized 80 M chitosan, in the presence of 0.5% heat-inactivated serum. These observations are quite different to those in PMN and monosodium urate crystals, which have a poor capacity for internalization while strongly activating PMNs, probably because of an autocrine effect [25]. It is highly likely that lipid mediators are involved in this autocrine effect. In our internalization assay, PMNs readily internalized zymosan, a yeast cell wall preparation that activates neutrophils. Because both 95 M and 80 M were internalized without activating neutrophils, our data demonstrate that internalization of a polysaccharide biomaterial does not automatically trigger degranulation.

Recently, PMNs were reported to express the mannose receptor [26], a receptor that is implicated in the internalization of chitosan by macrophages [27,28]. We provide evidence that monocytes internalize 80 M chitosan more readily than PMNs, suggesting that the molecular mechanisms involved in the internalization of 80 M chitosan by PMNs differ from those of macrophages. This does not imply a less important role of PMNs in chitosan-based wound healing. PMNs usually outnumber macrophages in certain phases of wound healing and can collectively synthesize large quantities of soluble mediators.

## Conclusions

In summary, 80 M chitosan is chemotactic for human PMNs but does not activate additional PMN effector functions such as degranulation and superoxide production. Because the beneficial therapeutic effects of 80 M chitosan are preceded by the recruitment of a significant number of PMNs, this chitosan-induced PMN phenotype could be associated with promotion of repair. Our observations also indicate that the degree of deacetylation is an important factor to consider in the use of chitosan as an accelerator of repair because PMNs exhibit a differential capacity to migrate towards 80 M and 95 M chitosan.

## Abbreviations

80 M: 80% deacetylated chitosan of medium viscosity; 95 M: 95% deacetylated chitosan of medium viscosity; cPLA2-α: cytosolic phospholipase A_2_-α; DDA: degree of deacetylation; FBS: fetal bovine serum; fMLP: *N*-formyl-methionyl-leucyl-phenylalanine; HTAB: hexadecyltrimethylammonium bromide; LTB_4_: leukotriene B_4_; MPO: myeloperoxidase; OA: osteoarthritis; PAF: platelet-activating factor; PDI: polydispersity index; PMN: polymorphonuclear neutrophil; RITC: rhodamine B isothiocyanate.

## Competing interests

The authors declare that they have no competing interests.

## Authors' contributions

PS and HG made equal contributions to the experimental aspects of this study. They performed the majority of the experiments. SM and DR made important contributions to the conduct of certain experiments as well as the interpretation of the data. MF, PP, and CH contributed to the design of the experiments and the interpretation of the data. MF wrote the manuscript, and PP and CH revised it. All the experiments were performed and supervised in MF's laboratory. HEG contributed to the interpretation of the data.
